# Activation of the Lectin Pathway Drives Persistent Complement Dysregulation in Long COVID

**DOI:** 10.1111/imm.70110

**Published:** 2026-01-25

**Authors:** Samuel B. K. Keat, Priyanka Khatri, Youssif M. Ali, Chanuka H. Arachchilage, Gregory Demopulos, Kirsten Baillie, Kelly L. Miners, Kristin Ladell, Samantha A. Jones, Helen E. Davies, David A. Price, Wioleta M. Zelek, B. Paul Morgan, Wilhelm J. Schwaeble, Nicholas J. Lynch

**Affiliations:** ^1^ Division of Infection and Immunity Cardiff University School of Medicine, University Hospital of Wales Cardiff UK; ^2^ Department of Veterinary Medicine University of Cambridge Cambridge UK; ^3^ Department of Microbiology and Immunology, Faculty of Pharmacy Mansoura University Mansoura Egypt; ^4^ Omeros Corporation Seattle Washington USA; ^5^ Department of Respiratory Medicine University Hospital Llandough Penarth UK; ^6^ Systems Immunity Research Institute, Cardiff University School of Medicine, University Hospital of Wales Cardiff UK

**Keywords:** complement, inflammation, lectin pathway, long COVID, therapeutics

## Abstract

Long COVID affects a substantial proportion of survivors of acute infection with severe acute respiratory syndrome‐associated coronavirus‐2 (SARS‐CoV‐2), who suffer a variety of symptoms that limit their quality of life and economic activity. Although the aetiology of long COVID is obscure, it appears to be a chronic inflammatory condition. Complement dysregulation is a prevalent feature of long COVID. Specifically, markers of classical, alternative, and terminal pathway activation are often elevated in patients with this condition. Here, we used a sensitive assay for mannan‐binding lectin‐associated serine protease‐2 (MASP‐2)/C1Inh complexes to analyse lectin pathway activation in a previously characterised cohort of patients with long COVID (*n* = 159) and healthy convalescent individuals with no persistent symptoms after infection with SARS‐CoV‐2 (*n* = 76). The data were combined with those from the most predictive complement analytes identified previously to delineate potential biomarkers of long COVID. MASP‐2/C1Inh complexes were significantly elevated in patients with long COVID (*p* = 0.0003). Generalised linear modelling further identified an optimal set of four markers, namely iC3b (alternative pathway), TCC (terminal pathway), MASP‐2/C1Inh (lectin pathway), and the complement regulator properdin, which had a receiver operating characteristic predictive power of 0.796 (95% confidence interval = 0.664–0.905). Combinations of the classical pathway markers C4, C1q, and C1s/C1Inh were poorly predictive of long COVID. These findings demonstrate that activation of the lectin complement pathway, which occurs upstream of the alternative and terminal pathways and can be inhibited therapeutically, is a salient feature of long COVID.

## Graphical abstract

The complement cascade, a series of blood proteins that assists in the recognition and neutralisation of pathogens, either independently or in conjunction with antibodies, is persistently activated in patients with long COVID. We found that blood plasma samples from patients with long COVID contained elevated concentrations of the mannan‐binding lectin‐associated serine protease‐2/C1Inh complex, indicating activation of the (antibody‐independent) lectin pathway, as well as downstream components of the complement cascade (iC3b, properdin, and the terminal pathway complex, TCC). These findings suggest that ongoing activation of the lectin pathway contributes to the pathogenesis of post‐acute sequelae of COVID‐19 (PASC). Inhibition of lectin pathway activation may be of therapeutic value in patients with PASC.

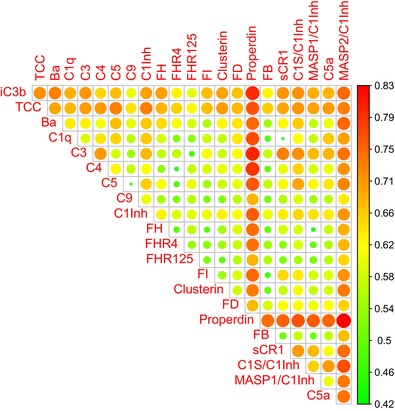

**Abbreviations**
MASP: mannan‐binding lectin‐associated serine protease
PASC: post‐acute sequelae of COVID‐19
SARS‐CoV‐2: severe acute respiratory syndrome‐associated coronavirus‐2

## 1 Introduction

A substantial proportion of survivors of acute COVID‐19 develop a chronic illness lasting longer than 12 weeks after infection with severe acute respiratory syndrome‐associated coronavirus‐2 (SARS‐CoV‐2), which is termed long COVID or post‐acute sequelae of COVID‐19 (PASC) [1, 2, 3]. According to a recent systematic review, 45% of individuals experience symptoms 4 months after infection with SARS‐CoV‐2, irrespective of the severity of acute COVID‐19 [4]. Long COVID manifests in various forms as a heterogeneous disease. Common symptoms include breathlessness and chest pain, cognitive defects (‘brain fog’), fatigue, and sensory dysregulation, all of which can have a substantial impact on quality of life [5]. Several mechanisms have been proposed to explain how acute infection develops into long COVID, including persistent exposure to viral antigens with or without an active reservoir of SARS‐CoV‐2, dysfunctional coagulation, microangiopathies and/or endothelial damage, and immune dysregulation [6, 7, 8, 9], which has further been linked with autoimmunity [10]. EBV reactivation has also been implicated in the development of long COVID [11]. Importantly, chronic inflammation appears to be a consistent feature of long COVID, irrespective of the underlying aetiology [12, 13, 14].

Complement activation is a driver of inflammation in many diseases, including acute COVID‐19 [15, 16, 17], where specific markers of each complement activation pathway predict disease outcome [15, 18, 19, 20, 21]. In a previous study, we detected activation products of the classical, alternative, and terminal complement pathways at significantly higher concentrations in plasma samples from patients with long COVID relative to healthy convalescent individuals with no persistent symptoms after recovery from infection with SARS‐CoV‐2. Plasma concentrations of some complement components and regulators also varied significantly between healthy convalescent individuals and patients with long COVID. Specific combinations of these markers were predictive of disease [22]. However, the only marker of lectin pathway activation included in this study was C1 inhibitor (C1Inh) in complex with mannan‐binding lectin‐associated serine protease‐1 (MASP‐1), concentrations of which were not significantly elevated in patients with long COVID.

Activation of the lectin complement pathway has been shown to be a therapeutically accessible feature of acute COVID‐19 [23, 24, 25]. The most informative markers of lectin pathway activation in these studies were the serine protease MASP‐2 and MASP‐2/C1Inh, a relatively stable covalent complex formed when MASP‐2 is activated and subsequently inactivated by its regulator, C1Inh. In the light of these findings, we used a recently developed bead‐based immunofluorescence assay to quantify plasma concentrations of MASP‐2/C1Inh in our previously characterised cohort of healthy convalescent individuals and patients with long COVID [22]. We found that plasma concentrations of MASP‐2/C1Inh were significantly higher in patients with long COVID relative to healthy convalescent individuals, indicating sustained activation of the lectin complement pathway. Importantly, the lectin pathway is activated upstream of the alternative and terminal pathways, suggesting a potentially key role in the pathogenesis of long COVID. In addition, we found that a nonredundant panel of four complement markers, including MASP‐2/C1Inh, iC3b, and the terminal complement complex (TCC), together with the positive regulator properdin, was optimally predictive of long COVID.

## 2 Methods

### 2.1 Participants Included in This Study

EDTA plasma samples were collected from age/ethnicity/sex/infection/vaccine‐matched healthy convalescent individuals (controls, *n* = 76) and patients with long COVID (cases, *n* = 159). All participants had a clinical history of acute COVID‐19 and direct molecular evidence of infection with SARS‐CoV‐2. Cases were diagnosed according to the National Institute for Health and Care Excellence (NICE) guideline NG188 (https://www.nice.org.uk/guidance/ng188). Eligible patients were men and nonpregnant women over the age of 18 years with no alternative explanatory disease and symptoms that persisted for at least 12 weeks after the initial diagnosis of acute COVID‐19. The most prevalent symptoms were breathlessness, fatigue, musculoskeletal problems, neuropsychiatric disturbances, and pain (Table 1). One persistent symptom was sufficient for the diagnosis of long COVID. All patients underwent a comprehensive medical evaluation, including chest radiography, electrocardiography, lung function tests (spirometry with gas transfer as indicated and measurement of exhaled nitric oxide), and standard blood tests (autoantibody screens, bone, liver, and kidney function, coagulation screens, full blood count, and markers of nutrition). In 47.3% of controls and 54.1% of cases, the index infection occurred > 2 years prior to sample acquisition, which was limited to a time window between February and October 2022. Symptoms were scored individually using a numeric self‐rating scale from 0 (no symptom) to 10 (worst possible symptom). Overall general health was scored similarly on an inverse scale from 0 (worst possible) to 10 (best possible). All participants reported age, ethnicity, and sex. Gender and socioeconomic status were not formally assessed in this study. Cohort demographics, symptomatology, and other key features are summarised in Table S1. A more detailed breakdown of vaccination history is presented for all participants in Table S2.

**TABLE 1:** Summary statistics for complement markers most predictive of long COVID.

Models	AUC	CI	*Z*	SE	*p*
MASP‐2/C1Inh	0.718	0.57–0.855	4.614	0.176	3.95E−06
MASP‐2/C1Inh + TCC	0.714	0.583–0.843	4.84	0.205	1.30E−06
MASP‐2/C1Inh + TCC + iC3b	0.742	0.6–0.868	4.867	0.216	1.13E−06
MASP‐2/C1Inh + TCC + iC3b + Properdin	0.796	0.664–0.905	4.934	0.231	8.04E−07

*Note*: ROC curves were generated using multiple GLMs for each complement protein. AUC statistics are shown for combined analytes with 95% confidence intervals generated from 2000 bootstrap replicates for each GLM.

### 2.2 Luminex Assay for MASP‐2/C1Inh and C1s/C1Inh Complexes

MASP‐2/C1Inh and C1s/C1Inh were quantified using a multiplexed bead‐based fluorescence assay, in which the capture antibody was directed against the serine protease, and the detection antibody was directed against C1Inh [24].

### 2.3 General Statistics

Group means were compared using unpaired *t*‐tests in Prism version 9.5.0 (GraphPad). Significance was assigned at *p* < 0.05.

### 2.4 Receiver Operating Characteristic Analysis

A series of generalised linear models (GLMs) using different combinations of protein measurement data with varying complexity were constructed via logistic regression using the base stats package in R, with a binomial model for error distribution and specified link function. Data were randomly split 70/30 into ‘training’ and ‘test’ sets to prevent overfitting and stratified to maintain case/control proportions, and ‘test’ data were reported as areas under the curves (AUCs). As the accuracy of each ‘test’ set prediction was equivalent to the true positive rate/false positive rate, the ‘predictive power’ or ‘reliability’ of each model was defined using the corresponding AUCs. GLMs containing three major confounders (age, sex, and BMI) were compared to GLMs containing the same confounders and each complement protein using the Delong test to quantify changes in model performance and define the effects of each analyte on the resultant AUCs. Protein concentrations were adjusted for age and sex and standardised to a mean of 0 and a standard deviation of 1 to maintain equal contributions of each protein in the analyses and prevent bias arising from proteins with wider concentration ranges, and analyses of unadjusted protein concentrations were used for comparison. A step Akaike Information Criterion (stepAIC) model was run to inform the best features to retain in the final model via iterative analyses of AICs. Models with fewer protein measurements were favoured to prevent overfitting and promote clinical utility. Sequential combinations of adjusted complement proteins were also included in a separate series of GLMs. The order of inclusion was informed by the ranking of the corresponding AUCs. Complement protein concentration distributions were compared using the Wilcoxon–Mann–Whitney test, and models were compared using receiver operating characteristic (ROC) curves, with 95% confidence intervals calculated via the default ‘bootstrap’ method across 2000 replicates for each AUC.

## 3 Results

In a previous study, we demonstrated that plasma markers used to quantify complement activation via the alternative (iC3b and Ba) and terminal pathways (C5a and TCC) were predictive of long COVID, with a combined AUC value of 0.785 established using GLMs [22]. However, the only marker of lectin complement pathway activation included in this study was MASP‐1/C1Inh, which was not significantly elevated in patients with long COVID. To reevaluate the conclusion that activation of the lectin complement pathway is not a key driver of disease, we used a recently developed and highly sensitive bead‐based immunofluorescence assay to quantify plasma concentrations of MASP‐2/C1Inh [24] in the same cohort of healthy convalescent individuals and patients with long COVID. We also used the same plasma samples to ensure direct comparability. The corresponding donor groups were closely matched for age (cases, median = 47 years; controls, median = 44.5 years), ethnicity (cases, white = 88.7%; controls, white = 84.2%), sex (cases, female = 78.6%; controls, female = 80.3%), time from infection to sampling (cases, 54.1% infected > 2 years before sample acquisition; controls, 47.3% infected > 2 years before sample acquisition), and vaccination status (cases, 94% vaccinated at least twice before infection; controls, 97% vaccinated at least twice before infection) (Tables S1 and S2). None of the major potential confounders in the matching process were significantly associated with donor group classification (Figure S1).

**FIGURE 1:**
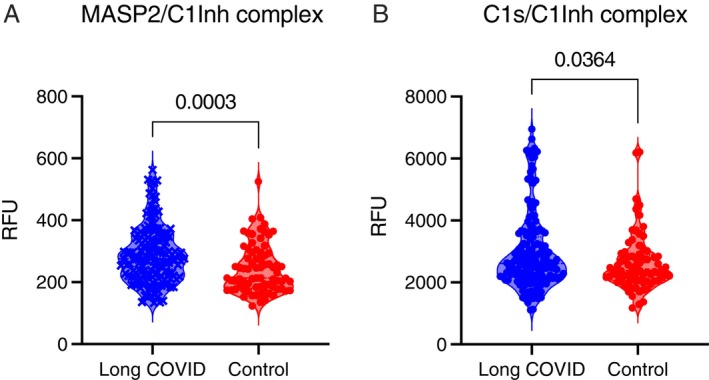
Plasma concentrations of MASP‐2/C1Inh are elevated in patients with long COVID. Plasma concentrations of MASP‐2/C1Inh (A) and C1s/C1Inh (B) in healthy convalescent individuals (*n* = 76) and patients with long COVID (*n* = 159). Significance was assessed using an unpaired *t*‐test.

**FIGURE 2:**
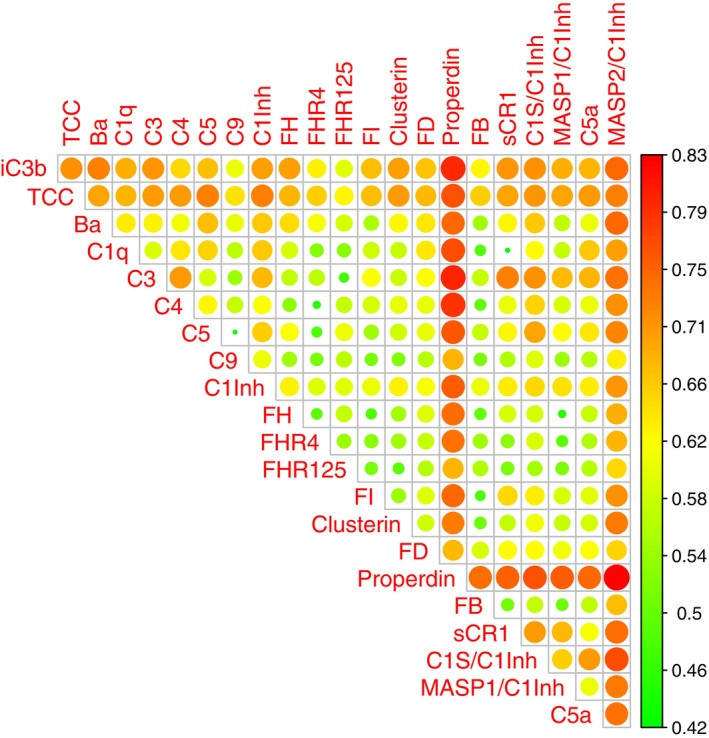
Individual plasma concentrations of MASP‐2/C1Inh, iC3b, TCC, and properdin are strongly predictive of long COVID. Heatmap showing pairwise comparisons of ROC area‐under‐the‐curve values obtained for different combinations of plasma complement biomarkers via logistic regression, adjusted for age, sex, and BMI.

**FIGURE 3:**
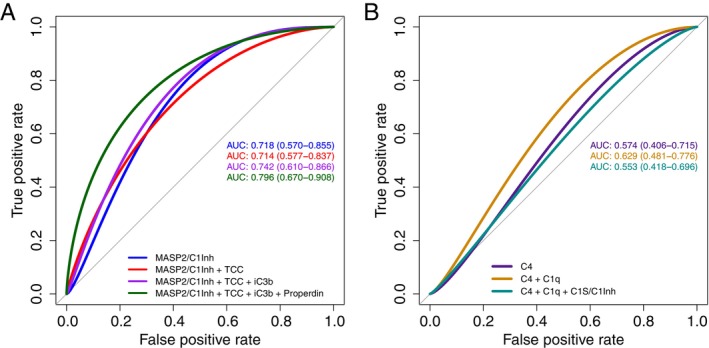
Combined plasma concentrations of MASP‐2/C1Inh, iC3b, TCC, and properdin are strongly predictive of long COVID. ROC curves for GLMs using the plasma concentrations of complement proteins and activation products to predict long COVID. ROC curves were generated using multiple GLMs for each complement protein. AUC statistics are shown for combined analytes, with 95% confidence intervals in parentheses calculated via the default ‘bootstrap’ method across 2000 replicates for each AUC. Markers were adjusted for age, sex, and BMI. Sequential combinations are shown for each of the top four ranked AUC statistics from complement GLMs (A), starting with the highest ranked AUC (iC3b). A combination of lectin and alternative pathway activation markers, together with plasma concentrations of properdin, yielded the highest positive predictive value. In contrast, subsets of classical pathway activation markers were poorly predictive (B), suggesting that the lectin pathway primarily drives downstream activation of the complement cascade in patients with long COVID.

**FIGURE 4:**
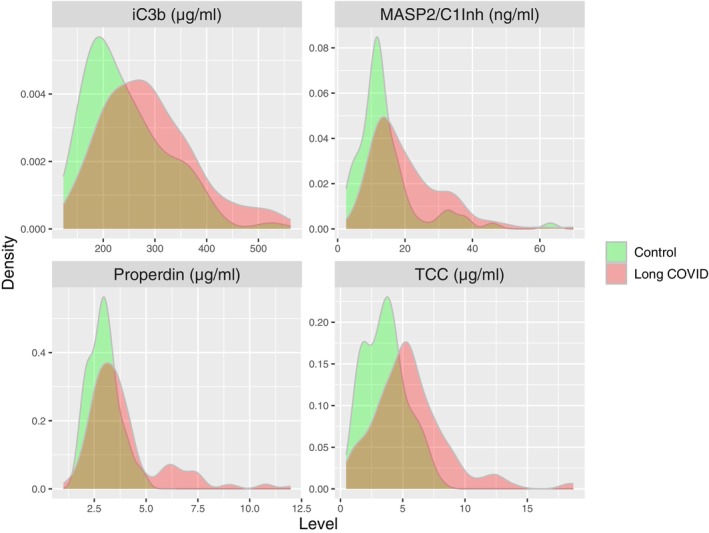
Plasma concentration distributions for MASP‐2/C1Inh, iC3b, TCC, and properdin in healthy convalescent individuals and patients with long COVID. Density plots showing the plasma concentration distributions for MASP‐2/C1Inh, iC3b, TCC, and properdin in healthy convalescent individuals (green, *n* = 76) and patients with long COVID (red, *n* = 159).

Plasma concentrations of MASP‐2/C1Inh and C1s/C1Inh, remeasured in parallel for validation, were significantly higher in patients with long COVID relative to healthy convalescent individuals (*p* = 0.0003 and *p* = 0.0364, respectively) (Figure 1). ROC analyses performed using GLMs spanning the entire dataset generated here and in the previous study [22] identified 10 complement markers with the potential to predict long COVID, four of which were activation markers (MASP‐2/C1Inh, iC3b, Ba, and TCC), and three of which were regulators of complement activation (C1Inh, properdin, and factor H [FH]). A pairwise comparison of the AUCs across all markers revealed the importance of MASP‐2/C1Inh, iC3b, TCC, and properdin (Figure 2). Marker combinations were then assessed for optimal predictive power using stepAIC. This approach identified an optimal set of four markers (MASP‐2/C1Inh, TCC, iC3b, and properdin), with an AUC of 0.796 (confidence interval = 0.664–0.905) (Figure 3a and Table 1), approximating the best set of eight markers reported previously [22]. Plasma concentration distributions for each of these four markers are shown in Figure 4.

We also considered the possibility that activation of the classical complement pathway was responsible for activation of the alternative and terminal pathways, evidenced by the elevated plasma concentrations of iC3b and TCC, respectively. Sequential combinations of the classical pathway markers C4, C1q, and C1s/C1Inh were poorly predictive of long COVID (Figure 3b and Table 2).

**TABLE 2:** Summary statistics for classical complement pathway markers of long COVID.

Models	AUC	CI	*Z*	SE	*p*
C4	0.574	0.406–0.715	4.636	0.172	3.56E−06
C4 + C1q	0.629	0.481–0.776	4.653	0.174	3.28E−06
C4 + C1q + C1s/C1Inh	0.553	0.418–0.696	4.661	0.181	3.14E−06

Collectively, these results suggest that activation of the complement cascade is triggered primarily via the lectin pathway rather than via the classical pathway, driving the production of terminal pathway components in patients with long COVID.

## 4 Discussion

There is ample evidence of complement activation during acute COVID‐19 [15, 16, 17, 18, 19, 20, 21]. All three activation pathways are thought to be involved in the response to infection with SARS‐CoV‐2. The classical pathway is activated by virus‐specific antibodies, whereas the lectin pathway is activated by viral subcomponents binding to innate recognition complexes or via direct activation of MASP‐2 by the nucleocapsid protein of SARS‐CoV‐2 [26, 27]. It is less clear how the alternative pathway becomes activated during acute COVID‐19. However, the generation of C3b via the classical or lectin pathways is likely to be amplified by the alternative pathway [28], which could also be activated by interactions between the virus and heparan sulphate on the surface of infected cells acting to dislodge the negative complement regulator FH [29]. Hypocomplementemia typically occurs in severe acute COVID‐19. Plasma concentrations of certain complement components and activation products can also predict clinical outcomes in patients hospitalised with acute COVID‐19 [15, 18, 19, 20, 21].

In a previous study, we demonstrated that activation products of the classical (C1s/C1Inh), alternative (iC3b and Ba), and terminal complement pathways (C5a and TCC) circulated at significantly higher concentrations in patients with long COVID relative to healthy convalescent individuals with no persistent symptoms after contemporaneous infection with SARS‐CoV‐2 [22]. Plasma concentrations of some complement components and regulators (C3, C4, C5, C9, C1Inh, FD, FH, and properdin) were also elevated in patients with long COVID, likely because they are acute phase reactants or, in the case of properdin, because the protein is released from neutrophil secondary granules in response to inflammation [30]. However, activation of the lectin pathway was assessed primarily via the use of a commercially available ELISA to quantify MASP‐1/C1Inh, plasma concentrations of which were not significantly elevated in patients with long COVID [22].

In the present study, we reexamined the role of the lectin complement pathway in the same cohort using a sensitive bead‐based immunofluorescence assay to quantify MASP‐2/C1Inh, which was multiplexed to enable the simultaneous measurement of C1s/C1Inh. We found that plasma concentrations of MASP‐2/C1Inh were significantly increased in patients with long COVID relative to healthy convalescent individuals with no persistent symptoms after contemporaneous infection with SARS‐CoV‐2 (*p* = 0.0003). A similar pattern was observed for C1s/C1Inh, consistent with the earlier results obtained via ELISA. Specifically, plasma concentrations of C1s/C1Inh were increased by 13% in patients with long COVID relative to healthy convalescent individuals in the present study (*p* = 0.0364) and by 15% in patients with long COVID relative to healthy convalescent individuals in the previous study (*p* = 0.0089) [22].

There are several explanations that could account for this apparent discrepancy between the lectin pathway activation markers MASP‐1/C1Inh and MASP‐2/C1Inh. Importantly, MASP‐2 is essential for lectin pathway activation, converting C4 to C4b and C4b‐bound C2 to C4b2a, the C3 convertase [31, 32]. MASP‐1 can accelerate this process but is not essential for lectin pathway activation. It is notable that elevated plasma concentrations of MASP‐1/C1Inh were reported in a previous study of patients with acute COVID‐19 [33]. The corresponding median values were 36.9 ng/mL in uninfected individuals and 51.5 ng/mL in patients with acute COVID‐19. The latter is remarkably similar to the median value observed among healthy convalescent individuals in our cohort (57 ng/mL), leading to the intriguing possibility that circulating MASP‐1/C1Inh concentrations remain high for a lengthy period after acute infection with SARS‐CoV‐2, irrespective of the development of long COVID.

A pairwise comparison of the AUCs obtained from univariate ROC analyses of each complement marker revealed the strong predictive value of measuring MASP‐2/C1Inh, iC3b, TCC, and properdin and the weak predictive value of measuring MASP‐1/C1Inh. In addition, the combination of MASP‐2/C1Inh, TCC, iC3b, and properdin yielded an AUC of 0.796 (0.664–0.905), suggesting a marker set that could form the basis of a manageable tool for the diagnosis of long COVID. MASP‐2/C1Inh, iC3b, and TCC are markers of complement activation via the lectin, alternative, and terminal pathways, respectively, whereas properdin is a positive regulator of the alternative pathway released from neutrophil granules in response to inflammatory stimuli, including complement activation products such as C5a. Plasma concentrations of circulating complement proteins were generally poorly predictive, as were markers of classical pathway activation, including C1s/C1Inh.

Collectively, these results indicate that activation of the lectin pathway contributes to the downstream activation of the alternative and terminal pathways observed in patients with long COVID. Activation of the lectin pathway might also explain the endothelial damage and persistent coagulopathy characteristic of long COVID [7, 8, 34]. The lectin pathway could be activated by aberrant glycocalyx patterns expressed on damaged endothelium [32, 35], resulting in activation of MASP‐2, which has factor Xa‐like activity and activates the coagulation system by cleaving prothrombin to form thrombin and factor XII to form factor XIIa [36, 37]. Accordingly, therapeutic inhibition of the lectin pathway has the potential to break the cycle of inflammation and ameliorate the clotting diathesis characteristic of long COVID.

## Author Contributions

S.B.K.K. analysed data and wrote the paper. P.K., Y.M.A., C.H.A., G.D., K.B., K.L.M., K.L., S.A.J., H.E.D., D.A.P., W.M.Z., B.P.M., and N.J.L. performed experiments and/or provided reagents/resources. H.E.D., D.A.P., W.M.Z., B.P.M., W.J.S., and N.J.L. conceived the project, led the study, supervised the work, and wrote the paper. All authors accepted responsibility for the work, approved the final draft of the manuscript, and concurred with the decision to submit for publication.

## Funding

This work was supported in part by the UK Dementia Research Institute (DRI), in part by the National Institute for Health and Care Research/UK Research and Innovation (NIHR/UKRI), grant numbers COV0170 (Humoral Immune Correlates of COVID‐19) and COV‐LT2‐0041 (The Immunologic and Virologic Determinants of Long COVID), and in part by Omeros Corporation, Seattle, USA. D.A.P. was further supported by the PolyBio Research Foundation (Balvi B43). W.M.Z. was supported by a Race against Dementia Alzheimer's Research UK Fellowship (520488).

## Ethics Statement

All participants provided written informed consent in accordance with the principles of the Declaration of Helsinki. Study approval was granted by the Cardiff University School of Medicine Research Ethics Committee (21/55) and by the Health Research Authority and Health and Care Research Wales (20/NW/0240).

## Conflicts of Interest

Y.M.A., W.J.S., and N.J.L. are consultants to Omeros Corporation, which is developing inhibitors of the lectin complement pathway. G.D. is employed by Omeros Corporation. The other authors have no conflicts of interest to declare.

## Supporting information


**Table S1:** Demographics, symptomatology, and other key features of healthy convalescent individuals and patients with long COVID.
**Table S2:** Vaccination status of healthy convalescent individuals and patients with long COVID.
**Figure S1:** Forest plot showing the regression coefficients (estimate values) of potential confounders of long COVID status with individual *p* values. ns, not significant.

## Data Availability

The data that support the findings of this study are available from the corresponding author upon reasonable request.
