# Supplementary material for: Activation of the Lectin Pathway Drives Persistent Complement Dysregulation in Long COVID
Source: Immunology. 2026 Jan 25;178(2):261–8. doi: 10.1111/imm.70110 (PMC13135881; doi:10.1111/imm.70110)
Supplement: Supplementary file 1 — Table S1: Demographics, symptomatology, and other key features of healthy convalescent individuals and patients with long COVID. Table S2: Vaccination status of healthy convalescent individuals and patients with long COVID. Figure S1: Forest plot showing the regression coefficients (estimate values) of potential confounders of long COVID status with individual p values. ns, not significant. [file IMM-178-261-s001.docx]

**SUPPLEMENTARY INFORMATION**

**Supplementary Table 1.** Demographics, symptomatology, and other key features of healthy convalescent individuals and patients with long COVID.

| Characteristic | Controls (*n* = 76) | Long COVID (*n* = 159) |
| --- | --- | --- |
| Age (years), median (range) | 44.5 (21–82) | 47 (20–83) |
| Female | 61 (80.3%) | 125 (78.6%) |
| Ethnicity/race  White  Black  Asian  Mixed ethnicity  Other | 64 (84.2%)  0 (0.0%)  8 (10.5%)  2 (2.6%)  2 (2.6%) | 141 (88.7%)  3 (1.9%)  8 (5.0%)  2 (1.3%)  5 (3.1%) |
| Body mass index >30kg/m^2^ | 24/69 (34.8%) | 77/155 (49.6%) |
| Coexisting conditions  Yes  No | 13 (17.1%)  63 (82.9%) | 53 (33.3%)  106 (66.7%) |
| Employment status  *Pre-COVID-19*  Employed  Employed, altered duties  Employed, sick leave  Unemployed  Retired  Student  *Post-COVID-19*  Employed  Employed, altered duties  Employed, sick leave  Unemployed  Retired  Student | 49 (64.4%)  0 (0.0%)  0 (0.0%)  0 (0.0%)  2 (2.6%)  4 (5.3%)  51 (67.1%)  0 (0.0%)  0 (0.0%)  0 (0.0%)  2 (2.6%)  2 (2.6%) | 136 (85.5%)  0 (0.0%)  0 (0.0%)  6 (3.8%)  9 (5.7%)  2 (1.3%)  68 (42.8%)  28 (17.6%)  31 (19.5%)  10 (6.3%)  10 (6.3%)  3 (1.9%) |
| Date of infection with SARS-CoV-2  March 2020 to August 2020  September 2020 to June 2021  July 2021 to October 2022 | 14 (18.4%)  22 (28.9%)  39 (51.3%) | 30 (18.9%)  56 (35.2%)  72 (45.3%) |
| COVID-19 vaccination status, median (IQR)  Number of vaccinations before infection  Total number of vaccinations | 3 (0–3)  3 (3–3) | 2 (0–2)  3 (3–4) |
| Symptoms^1^  Breathlessness  Fatigue  Musculoskeletal  Neuropsychiatric  Pain | 0 (0–0)  0 (0–2)  0 (0–0)  0 (0–0)  0 (0–0) | 4 (2–6)  6 (4–8)  3 (0–6)  3 (1–5)  4 (2–6) |
| Ability to maintain self-care | 0 (0–0) | 0 (0–3) |
| Ability to maintain daily tasks | 0 (0–0) | 6 (3–8) |
| Overall general health^2^ | 0 (0 to −1) | −4 (−2 to −6) |

^1^Difference in numeric rating scale score (0 = no symptom, 10 = worst possible symptom) before versus after COVID-19 (median ± IQR). ^2^Difference in numeric rating scale score (0 = worst possible, 10 = best possible) before versus after COVID-19 (median ± IQR).

**Supplementary Table 2.** Vaccination status of healthy convalescent individuals and patients with long COVID.

| Vaccinations | Controls (*n* = 67) | Long COVID (*n* = 148) | Total (*n* = 215) |
| --- | --- | --- | --- |
| 0 | 1 (1%) | 6 (3%) | 7 (3%) |
| 1 | 0 (0%) | 4 (2%) | 4 (2%) |
| 2 | 8 (12%) | 20 (13%) | 28 (13%) |
| 3 | 58 (87%) | 116 (81%) | 174 (81%) |
| 4 | 0 (0%) | 2 (1%) | 2 (1%) |

**Supplementary Figure 1.** Forest plot showing the regression coefficients (estimate values) of potential confounders of long COVID status with individual *P* values. ns, not significant.
